# Views of pregnant women and clinicians regarding discussion of exposure to phthalate plasticizers

**DOI:** 10.1186/1742-4755-11-47

**Published:** 2014-06-21

**Authors:** Sapna Sharma, Justin M Ashley, Alexandra Hodgson, Jeff Nisker

**Affiliations:** 1 Department of Obstetrics and Gynaecology, Schulich School of Medicine & Dentistry, Scientist Children’s Health Research Institute, Western University, London, ON, Canada; 2 London Health Sciences Centre, Victoria Hospital, Room E2-620E, 800 Commissioners Rd., E. London, ON N6A 5W9, Canada

**Keywords:** Pregnancy, Phthalates, Endocrine disruption, Household chemicals in pregnancy

## Abstract

**Objective:**

This study explores the views of pregnant women and clinicians regarding discussion of exposure to phthalate plasticizers during pregnancy, subsequent to the 2011 Health Canada ban of certain phthalates at a concentration greater than 1000 mg/kg in baby toys. This occurred with no regulation of products to which pregnant women are exposed, such as food packaging and cosmetics.

**Methods:**

Pregnant women, physicians and midwives were recruited through posters and pamphlets in prenatal clinics in Southwestern Ontario for a semi-structured interview. All interviews were audiotaped, transcribed, and subjected to rigorous qualitative analysis through a grounded theory approach, supported by NVIVO™ software. Themes emerged from line by line, open, and axial coding in an iterative manner.

**Results:**

Theoretical sufficiency was reached after 23 pregnant women and 11 clinicians had been interviewed. The themes (and subthemes from which they arose) were: Theme I-Information Provision (IA-Sources of Information, IB-Standardization, IC-Constraints, ID-Role of Government); Theme II-Risk (IIA-Significant Risk, IIB-Perceived Relevance, IIC-Reconciliation); and Theme III- Factors Influencing Level of Concern (IIIA-Current Knowledge, IIIB-Demographic Factors).

**Conclusion:**

To respond to the increasing media and research attention regarding risk of phthalates to women, and pregnant women in particular, national professional organizations should provide patient information. This could include pamphlets on what a pregnant woman should know about phthalates and how they can be avoided, as well as information to clinicians to facilitate this discussion.

## Introduction

Phthalates (diesters of 1,2-benzenedicarboxylic phthalic acid) are commonly present in food packaging, household cleaning products, and cosmetics [1,2]. In humans phthalates cross the placenta, resulting in fetal exposure that is closely correlated with maternal exposure [3], and have been found in amniotic fluid [4] and breast milk [5,6]. Increased maternal phthalates exposure has been associated with increased luteinizing hormone and decreased free testosterone [5], shortened anogenital distance [7], reduced penile size and incomplete virilization [5,8,9], altered semen quality [10,11], and preterm birth [12]. Exposure of pregnant women to phthalates has also been associated with adverse childhood behavior and executive functioning [13]. Braun, Sathyanarayana and Hauser [14] in their review of “phthalate exposure and children’s health” also draw attention to the correlation of urinary phthalate metabolite concentrations in physical growth [15,16] and allergic phenomena [17-20] in children. In animal models, phthalates have been associated with impaired spermatogenesis [21], cryptorchidism [22-24], hypospadias [24,25] and reduced male fertility [22].

In 2006, the Center for the Evaluation of Reproductive Risks to Humans completed an analysis of the developmental and reproductive risks to humans of several phthalate esters, raising concern and stressing the need to evaluate more precisely the risks associated with human exposure [26]. The European Union began regulating the concentrations of certain phthalates in children’s toys in 1999, and eventually made their policies stricter by implementing a ban in 2005 [27]. The United States began regulating phthalates in children’s toys in 2008 [28]. Finally, in 2009, Canada placed phthalates on the Hazardous Products Act, restricting the concentration of certain phthalate esters to “no more than 1000 mg/kg in the vinyl of all children’s toys and child care articles” [29].

Recently, Zimmer and coauthors (2012) [30] summarizes the opinions of a group of experts in attendance at the Health and Environment Network Conference in Belgium in 2010. The goal was to support informed policy making by facilitating the availability of relevant knowledge on phthalate exposure. Following expert opinion on relevant issues, intrauterine exposure and reproductive toxicity were prioritized as an area of further research. This group also encouraged policy actions, including monitoring, awareness raising, and restricting activities. This implies that although additional research is needed to further elucidate the risk that phthalates pose to human health, the current scientific knowledge is sufficient for using the precautionary principle to take policy actions such as restrictions and banning. Finally, they encouraged political action to protect groups at high risk of intrauterine exposure [30].

Along with these regulations and bans came media interest. As early as 2010, a CNN program on “5 toxins that are everywhere,” advised viewers to “protect yourself” from phthalates [31]. In 2011 an article in the popular women’s magazine “Glamour” cautioned against “The New Toxic Threats to Women’s Health”, and listed phthalates as one of the five household chemicals to avoid [32].

Despite the Canadian ban on certain phthalates in children’s toys, as well as media concern available to pregnant women in Canada, no similar ban has been proposed for products containing phthalates to which pregnant women are exposed. As such, this study was conducted to explore the views of pregnant women and clinicians regarding information on phthalate exposure, in order to elucidate any deficiencies in this process as well as to develop possible solutions.

## Methods

### Recruitment

Pregnant women in Southwestern Ontario were invited through posters and pamphlets in prenatal clinics, prenatal education classes, and prenatal fairs. Obstetricians, family physicians, and midwives in Southwestern Ontario were recruited through email, posters and pamphlets. Research ethics approval was obtained through the Western University’s Health Science Research Ethics Board (17406E).

On the pamphlets was a paragraph, which provided general information on phthalates:

“Phthalates are compounds that are used to make plastics flexible in their final applications. They are used in floor tiles, clothes, medical supplies, toys, food packaging, and personal care products. These compounds have also been shown to leach out of various products, and are also present in appreciable amounts in our environment….. mimic naturally occurring hormones in the body, interfering with the endocrine system to produce adverse developmental and reproductive effects. However, the full range and extent of these effects have not yet been identified.”

### Interviews

The 20–40 minute interviews were semi-structured, conducted by SS, JA and AH, and the participants were encouraged to provide their opinions at length. The non leading potential interview prompts that could be offered to the pregnant women were the same as those that would be offered to the clinicians (Table 1). In our research, participants were encouraged to speak freely for as long as they wanted on phthalate exposure in pregnancy and the prompts were only used if the participant’s opinions in the desired areas were not already expressed. The interviews were audiotaped and transcribed verbatim, including pauses and notes on the emotional tone of the spoken text. All identifying information was removed from the transcripts. The audiotapes were erased following transcription of the interviews.

**Table 1:** Potential interview prompts to be used in interviews of pregnant women and clinicians

**1**	Are you aware of any everyday environmental exposures that could impact the health of your child during pregnancy?
**2**	Are you aware of the government warnings regarding Bisphenol A and baby products like rubber duckies and baby bottles?
**3**	Could you tell me what sources of information you tend to use in regards to your children’s health?
**4**	What do you believe is the role/responsibility of your obstetrical care provider when it comes to your health and the health of your child during pregnancy?
**5**	Is there a point at which you think various parties should be providing you or the general public with information regarding potential risk? If so, who? And why? Please expand.
**6**	What do you believe is your role in obtaining information about potential environmental risks to you and your child during pregnancy?

### Analysis

The transcripts were entered into NVivo™ software (QSR International Proprietary Limited Company Ltd, Doncaster, Victoria, Australia), which allowed the researchers to manage the large quantities of text for qualitative analysis. This software also assisted in organizing text and searching for words. The interview transcripts underwent rigorous analysis using the analytical techniques of grounded theory methodology [33,34], which were specifically designed for analyzing complex social processes [35]. The analysis was organized into three phases: open, axial, and selective coding [33,34].

In open coding, the data were read and then fractured by identifying chunks of data that relate to a theme or idea. In axial coding, similar themes were organized into conceptual categories. In selective coding, a core concept was identified as the central theme of the study and the conceptual categories organized in relation to the core concept. Each stage of the coding process (open, axial, and selective) provided a set of categories that could be used to explore the emerging themes of the views of pregnant women and clinicians regarding the discussion of phthalate exposure in pregnancy. The axial level of coding built on the open coding level, incorporating categories and creating new ones [33,34].

Theoretical sampling was maintained throughout the analysis, and continued for as long as new themes and relationships were discovered in the data [33,34]. By using a process of constant comparison between the emerging theory and codes with those that had come before, as recorded in ongoing research memos, a grounded theory rather than a description of themes was generated [33,34].

## Results

**Figure 1:**
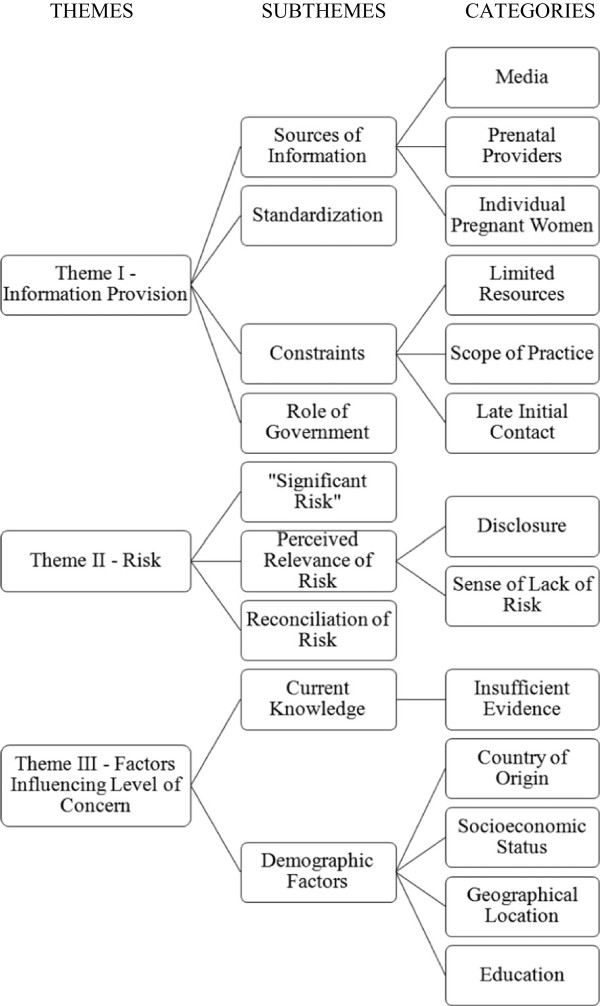
Themes and the categories and subthemes from which they arose.

Theoretical sufficiency [36] was reached after the transcripts of 23 pregnant women and 11 clinicians (6 obstetricians, 3 family physicians, 2 midwives) had been analyzed. Comments of pregnant women and obstetrical care providers were grouped together to form 13 categories based on content they shared (Figure 1). These categories were then grouped together to form subthemes, which were then combined to form the following themes: Theme I- Information Provision, Theme II- Risk, and Theme III- Factors Influencing Level of Concern (Figure 1).

### Theme I - Information provision

From the subtheme of Sources of Information, pregnant women and clinicians commented on who should be responsible for providing information on the risk of phthalates exposure in pregnancy. For example, Pregnant Woman 3 felt that: “The healthcare provider’s role is to…keep…you healthy and unfortunately that’s the only person that’s going to be able to do it”. Obstetrician 5 offered a different position:

…I don’t think you should put the onus on the family doc per se.… they’ve got so many things on their plate they can’t be aware of any, sort of, potential exposure or risk factor with pregnancy in the first trimester -OB5

Pregnant Women 4 and 6 stressed the importance of the pregnant woman finding information for herself:

…to an extent I believe you should be researching it yourself because your obstetrician doesn’t have time to know… everyday products that you are using, which brand of product, and to be researching everything herself…she should have a knowledge, but she doesn’t have knowledge on every single product -PW4

Well, it’s, I mean, its parents’ responsibility to try, you know, to look out for their children and do the best they can to keep them healthy, particularly in the womb when they’re most vulnerable. -PW6

Family Physician 1 highlighted the potential role of the internet:

the vast majority [of patients] still come with the confidence in their doctor to give them a kind of perspective … [but] things on the internet–um-can be-um inflamed or minimized. –FP1

Pregnant Woman 13 alluded to the overlap between information provision from a physician, as well as from the Internet:

…I’m usually…happy with what the doctor says but if it was something that I’d read on the Internet then…that’s something I would ask the doctor about it, but if the doctor had said it in the first place I wouldn’t go check on the Internet -PW13

Family Physician 2 commented:

…family physicians and midwives … have more access to patients at an earlier stage of pregnancy. –FP2

Midwife 2 alluded to the possibility of incorporating discussion of phthalates into post-partum care, as a means of introducing this information prior to subsequent pregnancies:

…we don’t see a lot of people, but actually we can do it postpartum … when we’re doing our discharge visit about when you can, when you want another baby, folic acid and all that, um, so we can definitely put a plug in there too. -MW2

The Theme of Information Provision also arose from the subthemes of Standardization, Constraints, and the Role of the Government. With respect to issues of Standardization, Obstetrician 1 stated that:

…I think standardization could be done… combined clinics where some of your prenatal clinics are as a group first antenatal visits you could lump people in 8 weeks to 10 weeks, or make it a month span, …go over all the prenatal stuff… -OB1

Alluding to perceived constraints on information provision, Obstetrician 2 and Midwife 1 felt that:and:

…only 50% of the pregnancies are planned, you do need to target the fertile, um, population if you want to do risk avoidance in pregnancy … the harmful effects are greatest in the period the baby forms their organs, um, you should really tackle them before they’re six weeks pregnant and the first two weeks before they miss a period are crucial. So they think they’re pregnant and they can’t get an appointment with their family doctor until two weeks later, so by the time they see the family doctor organogenesis is complete and, um, they are too late. –OB2

…we need a evidence-based pool of information that we can actually say to women, here’s the evidence on this… I think it’s always a difficult thing to, you know, to make recommendations to women if you don’t really have a solid background… –MW1

Finally, with respect to the role of the government in information provision, Pregnant Woman 1 felt that:while Obstetrician 1 stated:

Well, I think that there should be studies going on, and government giving money to studies to make sure we can reduce the risk of any problems down the road …. –PW1

… the government should have some … sort of web page that they can list these things on if people want to know what’s in what and everything… -OB1

### Theme II - Risk

Theme II was derived from the 3 subthemes: Significant Risk, Perceived Relevance of Risk, and Reconciliation of Risk. The subtheme of Significant Risk was contributed to by Pregnant Woman 5, who stated:

Risk is risk, whether it’s great or small, right… and babies are innocent so they deserve a fair shot, you know… I know it might seem a small risk, but that one small risk might be that risk that you didn’t have to take… -PW5

Regarding the subtheme of Perceived Relevance of Risk, Obstetrician 1 found:and Obstetrician 4 stated:

…it amusing the public fascination with this and Bisphenol A and so forth, yet people smoke throughout their pregnancies, yet people who drive their SUVs with one person in the car with them, like, like when you think about the volume of exposure there are some things that are exposing us to much more risk without question. –OB1

…I’m not going to pick on one particular item that has the potential esoteric, ah. associated risk factor that’s not proven through science, cause honestly that can open up a kettle, ah can of worms… -OB4

Pregnant Woman 21 explained how she perceives risk when it comes to the availability of products in the Canadian market:

…if there’s a product in the shelf in Canada I would think it’s safe to use. Like I shouldn’t have to take it [laughs] everything I buy and say, is this safe or what. I don’t and maybe I should question more but I don’t. I just take it for granted… –PW21

Regarding the subtheme of Reconciliation of Risk, Obstetrician 1 acknowledged that while there may exist a risk of phthalate exposure in pregnancy, there may be greater risks associated with exposure to alternate products:

….there may be a risk but then the benefits outweigh the risks. I don’t know for the soft plastics, like, soft plastics, but we can give our kids wooden toys but they’re probably laced with lead or arsenic or something. -OB1

Pregnant Woman 16 commented on how she reconciles risk:

Ah, you know what, I find, you know, in this day and age that just about everything could be of concern anyway, and you know, the things, you know, we don’t know, I guess, kinda of hard to avoid everything, I guess, especially if it’s in your, you know, daily living, you know, the exposures, so, kinda hard. -PW16

Whereas Pregnant Woman 1 reconciliation of risk differs:

…we don’t buy plastic water bottles anymore, we switched to the keep clean containers, the hard, hard stainless steel”. –PW1

### Theme III - Factors influencing level of concern

The third theme emerged from the subthemes of Current Knowledge and Demographic Factors. The subtheme of Current Knowledge was derived from comments such as that of Obstetrician 2 who stated:

If I knew what products contain … phthalates…and if I knew what to offer my patient and to how to avoid those, then I probably would give more information. –OB2

And Obstetrician 5 who commented:

…I think presently there isn’t that much data on environmental risks and early fetal development so I’m not sure that you can talk in detail about environmental risks… -OB5

The second subtheme, Demographic Factors, was derived from comments that formed the categories of Country of Origin, Socioeconomic Status, Geographic Location, and Education, respectively. For example, Obstetrician 2 commented that:

…I still don’t know what to tell my patient. So I can’t say, you know, buy this kind of pillow but don’t buy that, or buy this and don’t buy that and then the second thing is that I think it’s very prevalent in the affordable products and people often do not have the financial resources to go any other way. -OB2

Pregnant Woman 6 shared:

…it concerns me being near the Chemical Valley, you hear about the reserve out there, that there’s more girls being born than boys, but we live in Camlachie, which is part of the reason we moved a little further away… -PW6

Family Physician 1 commented:

I would actually say that, um, educated women, I mean with a higher level of education, probably have more questions and concerns about… risks…Um, so they are concerned but just in a broad sense I would say, that ah, that sometimes a little bit of knowledge then increases your anxiety about what potential you could put your child at risk for. -FP1

Pregnant Woman 12 described how her professional training has influenced how she is able to appraise risk:

[Being a nurse I’m] used to doing research papers and term papers so you…know where to find the information, whereas maybe your lay person might not know where to go to find information, might just plain Google and not realize some stuff that’s not as relevant. -PW12

## Discussion

The main research findings centered on the comments of research participants in both groups, pregnant women and clinicians, indicating their desire for more information on the effect of phthalate plasticizer exposure on the developing fetus, so that informed choice could be made regarding whether there is a need to avoid phthalate plasticizers in pregnancy, and, in the pregnant women group, how to avoid them regardless of certainty of the human and animal research. The comments of the clinicians indicated they were not willing to discuss phthalate exposure with their patients until their professional organizations provided a clinical practice guideline precisely outlining the risks of phthalate exposure in pregnancy, while the pregnant women wanted information either from their clinicians or from the government as soon as possible regarding how to avoid phthalate plasticizers. Pregnant women cared less about the certainty of the risks of phthalate plasticizers then the clinicians, and were more worried about the condemnation of phthalate plasticizers in the media.

Given the animal [21-25] and human [3-11] research suggesting adverse outcomes associated with prenatal phthalate exposure, and the recent media attention encouraging women to avoid phthalates in household products, the discussion of phthalate exposure in pregnancy can be important to pregnant women. However, as indicated in Theme I - Information Provision, prenatal care providers (obstetricians, family physicians, midwives) in our research did not perceive engaging in the discussion of phthalate exposure in pregnancy as relevant. The reasons provided include: (i) limited resources (e.g., interaction time); (ii) limited scope of practice; and (iii) late initial contact points. Sathyanarayana and coauthors (2012) [37] suggest similar lack of education and interest among clinicians caring for pregnant women and women who are contemplating pregnancy regarding other environmental exposures and provide tools to assist clinicians in counseling their patients. These discussions could incorporate Theme III – Factors Influencing Level of Concern found in our research as women could share their perceptions, including those derived from media portrayal of phthalates.

Considering Theme II - Risk, it is possible that clinicians find it difficult to discuss risks of phthalate exposure because the research on humans is not definitive and no firm clinical practice guidelines currently exist in most countries. An American College of Obstetricians and Gynecologists and American Society for Reproductive Medicine Committee Opinion on exposure to toxic environmental agents does include endocrine disrupters [38]. Furthermore, as prenatal care generally occurs following the period of organogenesis, the time during which the embryo is most susceptible to the effects of phthalate exposure [23], so it is important that women’s health primary care clinicians who confine their practice to the care of pregnant women or general obstetrics and gynecology frequently would not be able to provide counseling regarding phthalate exposure until it is potentially too late. Thus, it is important that primary care physicians, whether or not they care for pregnant women, be educated regarding and be able to discuss phthalate exposure preconception and during pregnancy.

For pregnant women, the lack of a ban or even a caution label on household products containing phthalates makes appreciation of risk complex. It is important that education and discussion of phthalate exposure related to pregnancy occur, even though making a direct causal relationship remains difficult due to incomplete understanding of the kinetics of phthalates, likely due to the exposure of pregnant women to a mixture of chemicals [39], for example, phthalates in cosmetics usually coexist with parabens [5].

To raise awareness regarding the potential risks of phthalate exposure to pregnant women, pamphlets and online resources such as those prepared by the Departments of Obstetrics and Gynecology at the University of California San Francisco and the University of Florida, should be included in Canadian prenatal care and made widely available to pregnant women. Given that family physicians care for women preconceptually and early in pregnancy, it is essential to have pamphlets available in their offices. Further, information on potential phthalate exposure should be included in continuing professional development programs. For example, a continuing professional development tool entitled “Environmental Impacts on Reproductive Health” has been developed by the Association of Reproductive Health Professionals (ARHP) and Planned Parenthood Federation of America (PPFA) [40], and an editorial entitled “Potential Toxicity of Synthetic Chemicals: What You Should Know About Endocrine-Disrupting Chemicals” appeared in the American Family Physician as a resource for clinicians [41]. Perhaps Canadian organizations with a vested interest in Reproductive Health, including, the Society of Obstetricians and Gynaecologists of Canada, Canadian College of Family Physicians, and the Canadian Association of Midwives, could consider producing similar materials to enable clinicians to discuss with their patients the risks and potential avoidance strategies phthalate exposure in pregnancy.

Qualitative research is designed to delve deeper into complex issues than is possible through surveying larger numbers using quantitative approaches [35]. In rigorous qualitative research we aim to present the comments of the research participants in an open and balanced manner, with the understanding that the findings may not be generalizable beyond the research participants, and may or may not be dependent to geographic location and socioeconomic or other factors. Indeed qualitative research findings should be considered as co-constructed [42] between the research participants and the research team. The results represent themes co-constructed from the research participants comments but do not necessarily capture all individual perceptions. Due to the methodological limitations of constructivist grounded theory, this research is not generalizable beyond the pregnant women interviewed. Readers are encouraged to consider the similarities and differences of their experiences with those shared in this study. Further research is needed to explore women’s experiences with household chemical risks in other contexts, cultures, and geographic locations.

## Conclusion

The increasing research and media attention regarding the risks of phthalates in pregnancy may create concern for pregnant women in Canada, thus prompting the need to address such concerns in clinician-provided information. The Society of Obstetricians and Gynaecologists of Canada, the Canadian College of Family Physicians, and the Canadian Association of Midwives should consider including information on phthalate exposure in pregnancy in their patient information strategies, and continuing professional development opportunities.

## Competing interests

The authors declare that they have no competing interests.

## Authors’ contributions

The design of this research and the writing of the manuscript was performed by by SS and JN. The interviews were conducted by SS, JMA and AH. The analysis was performed by SS and JN. All authors read and approved the final manuscript.
